# Advances in Food Allergy Immunotherapy: Current Strategies and Role of Antibodies Isotypes

**DOI:** 10.3390/cells14120900

**Published:** 2025-06-14

**Authors:** Yolanda Garcia-Carmona, Maria A. Curotto de Lafaille

**Affiliations:** 1 Department of Immunology and Immunotherapy, Precision Immunology Institute (PrIISM), Icahn School of Medicine at Mount Sinai, New York, NY 10029, USA; maria.lafaille@mssm.edu; 2 Division of Allergy and Immunology, Department of Pediatrics, Jaffe Food Allergy Institute, Icahn School of Medicine at Mount Sinai, New York, NY 10029, USA

**Keywords:** food allergy, allergen-specific antibodies, anti-IgE immunotherapy, oral immunotherapy (OIT), biomarkers

## Abstract

Food allergies result from dysregulated immune responses to dietary antigens. IgE antibodies are key in triggering allergic reactions through binding to high-affinity receptors on mast cells and triggering mast cell activation when crosslinked by allergens. In contrast, IgG antibodies—particularly IgG4—are linked to immunomodulation and tolerance. Allergen-specific memory B cells, especially IgG1+ cells, undergo class-switching to IgE, and IgE plasma cells underlie allergy persistence. Although there is no cure, allergen-specific immunotherapy (AIT) aims to achieve sustained unresponsiveness by gradually increasing allergen exposure. Oral immunotherapy (OIT), a form of AIT, induces a shift from a TH2-skewed response to a more regulated immune profile, characterized by a switch from IgE to IgG4 and IgA isotypes. This review outlines current insights into AIT’s cellular and humoral mechanisms, with implications for improving long-term outcomes and developing predictive biomarkers.

## 1. Introduction

Food allergy (FA) is a serious public health concern, affecting over 220 million individuals worldwide, including approximately 33 million people in the United States—5.6 million are children [1]. The majority of food allergies emerge in early childhood, with approximately 6% of children experiencing allergic reactions within their first three years, mostly to cow’s milk, eggs, and peanuts [2]. Nowadays, the prevalence of food allergies is increasing globally. Because it develops so quickly, it is unlikely that genetics alone can explain it. Instead, various environmental factors have been implicated in modulating allergy prevalence, including dietary changes, urbanization, pollution, microbial and endotoxin exposure, farming environments, and raw milk consumption [3]. A limited group of foods accounts for most food allergies, notably milk, egg, peanuts, tree nuts, fish, shellfish, soy, wheat, and sesame [4]. While allergies to milk and eggs tend to be outgrown by school age, peanut, nut, and seafood allergies tend to persist for life [5,6,7].

Currently, there is no cure for food allergies; allergy care strategy relies on eliminating trigger foods and controlling symptoms. However, while allergen avoidance and pharmacotherapy (e.g., antihistamines, adrenaline, corticosteroids) are effective, patients are still at risk of severe reactions from accidental exposure. Oral immunotherapy (OIT) is effective in increasing thresholds of tolerance and can, in some cases, lead to sustained unresponsiveness (SU), but it often causes allergic reactions, and its benefits may disappear when allergenic food consumption is interrupted [8]. More recently, biologicals offer hope for modulating the disease, but their ability to change the course of the disease is still not known [9]. Therefore, it is essential to gain a deeper understanding of the mechanisms underlying food tolerance and the development of food allergies.

## 2. Humoral Response

### 2.1. Immunoglobulin E (IgE) in Immunity: Biological Humoral Response

IgE-mediated food allergy involves the production of allergen-specific IgE antibodies that activate mast cells (MCs) and basophils upon ingestion [10]. IgE evolved in early mammals to defend against parasites, venoms, and toxins [11], but it is also key in allergic reactions and chronic inflammation, acting as the primary antigen-specific trigger of allergic responses [12]. Structurally, IgE consists of two light and two heavy chains with four constant domains (Cε1–Cε4) and no hinge region, similar to IgM [13]. It exists in both secreted and membrane-bound forms, the latter functioning as the B cell receptor (BCR). IgE does not activate complement and primarily exerts its effects through the high-affinity IgE receptor (FcεRI, Kd ~1 × 10^−10^ M)—mostly expressed on basophils and MCs [14]—and the low-affinity IgE receptor (FcεRII or CD23)—mostly expressed on B cells [15]. These receptors have distinct roles that are critical for mediating allergen-driven immune responses [16]. The binding of IgE to FcεRI is the trigger of allergic reactions. Despite its potent activity, IgE is tightly regulated [17,18], having the lowest serum levels among immunoglobulins (50–200 ng/mL vs. 1–10 mg/mL) and a short half-life of ~2 days—though this extends to several weeks when bound to FcεRI [19].

### 2.2. Allergen-Specific IgE Antibodies

**Figure 1:**
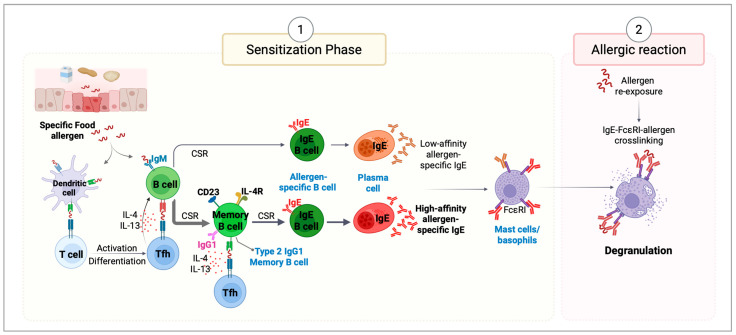
Mechanisms of allergic reactions. (**1**) Sensitization Phase. Epithelial cells respond to external insults by producing alarmins (IL-25, IL-33, and TSLP)—key regulators of type 2 immunity. These signals activate dendritic cells (DCs), enhancing their ability to promote Th2 responses and stimulate the production of IL-4 and IL-13. Once allergens cross epithelial barriers, they are captured and processed by DCs, which migrate to draining lymph nodes to present allergen-derived peptides via MHC class II to naïve T cells. Depending on the surrounding cytokine and co-stimulatory signals, these T cells differentiate into T helper type 2 (Th2) or T follicular helper (Tfh) cells. Th2 cells produce type 2 cytokines, including IL-4, IL-13, IL-5, and IL-9, which orchestrate allergic inflammation. Tfh cells support primarily sequential IgE class switch recombination (CSR) in B cells, promoting their differentiation into plasma cells that secrete allergen-specific IgE antibodies. These IgE antibodies circulate in the bloodstream and bind to FcϵRI receptors on mast cells and basophils, priming them for future allergen exposure. (**2**) Allergic Reaction Phase. Upon subsequent re-exposure to the same allergen, it binds to the antigen-specific IgE bound to mast cells and basophils. Allergen cross-linking of IgE on mast cells triggers rapid degranulation and release of inflammatory mediators. This cascade triggers local and systemic allergic symptoms, contributing to the clinical manifestations of allergic reactions.

Food allergies arise when immune tolerance to dietary antigens fails, resulting in the generation of high-affinity IgE antibodies that initiate allergic responses [2]. Once allergens cross the epithelial barrier, dendritic cells present them to naïve T cells, promoting differentiation into CD4+ Th2 (T helper 2) and Tfh2 (T follicular helper 2) cells, which drive IgE class switching in B cells via IL-4 and IL-13. This results in the generation and circulation of allergen-specific IgE antibodies [20,21] during the initial and asymptomatic stage (sensitization) (Figure 1). In this phase, the immune system recognizes a specific allergen and generates a response in which IgE antibodies are produced and will prime mast cells for future allergic reactions. Upon subsequent allergen exposure, MCs and basophils degranulate, releasing histamines, cytokines, leukotrienes, and prostaglandins, causing immediate (within minutes of allergen exposure) allergic symptoms and promoting inflammation. This quick response is driven by the high affinity of IgE for FcεRI [22] and the high proportion of allergen-specific IgE in atopic individuals, despite its low serum concentration [11]. Mast cell activation may cause symptoms that vary from skin reactions like hives to severe, potentially fatal, anaphylaxis [2]. Furthermore, IgE sustains the active effect of allergen-specific Th2/Tfh cells by presenting antigen-IgE complexes bound to IgE receptors. This increases the antigen presentation efficiency 10–100 times compared to standard pinocytosis-dependent pathways. This mechanism effectively activates allergen-specific T cells, even at low allergen exposure, as seen in respiratory allergies [3].

### 2.3. IgG and IgA Biological Humoral Response

IgG is the most abundant antibody in human serum (comprising 10–20% of total plasma immunoglobulins), crucial for neutralizing pathogens, promoting phagocytosis, and activating complement [23]. Humans produce four IgG subclasses (IgG1–4), each with unique immune functions [23]. IgG1 is the most abundant IgG subclass in human serum. Both IgG1 and IgG3 are mainly induced by protein antigens, whereas IgG2 predominantly targets repetitive, T cell–independent polysaccharides. IgG4, unlike other subclasses, has limited effector functions, such as complement-dependent cytotoxicity or ADCC (antibody-dependent cell-mediated cytotoxicity) [24]. It is also associated with tolerance, particularly in response to non-microbial antigens like food, and, as well as for IgE, IgG4 production relies on help from Th2/Tfh2-like cells [24,25]. As discussed later, there is strong evidence suggesting that food-specific IgG antibodies contribute to protection against food-induced reactions.

IgA dominates mucosal surfaces and supports immune tolerance to microbiota by excluding antigens and shaping microbial communities, while also exerting protection against external antigens [26]. It exists as monomeric IgA in serum and dimeric secretory IgA in mucosa, and, despite being the most produced antibody, serum IgA has a short half-life (about three days) [27]. IgA functions through various receptors to mediate immune regulation, including pIgR for transport, FcαRI (CD89) on immune cells, and several other receptors like CD71, ASGPR, Fcα/μR, FcRL4, and DC-SIGN/SIGNR1 [28]. Mucosal IgA is capable of recognizing antigens with high affinity (to neutralize microbial toxins and invasive pathogens) and low affinity (to confine commensals in the intestinal lumen) [29]. Several studies have linked low IgA levels with increased risk of atopic disorders. For instance, reduced IgA in the saliva has been associated with allergic sensitization, rhinitis, and eczema, while higher levels appear protective in sensitized infants [30,31,32,33]. Elevated fecal IgA is similarly correlated with a lower risk of atopy [34]. Together, these findings indicate that deficient IgA responses may contribute to allergic disease development.

## 3. Cellular Response

### 3.1. Isotype Class Switching, Affinity Maturation, and IgE Production

Class switch recombination (CSR) occurs in mature B cells within secondary lymphoid organs after antigen exposure, involving activation-induced cytidine deaminase (AID)-mediated recombination between switch (S) regions upstream of constant (C) genes [35]. To undergo class switching to IgE, B cells require CD40 engagement by CD4^+^ T helper cells along with signaling through the interleukin-4 receptor (IL-4R), triggered by T cell-derived IL-4 or IL-13 [18,36]. This activates the transcription factor STAT6, promoting the expression of genes encoding for CD23, IL4R, and IL13R, as well as the noncoding germline Cε transcript (*Ighe* in mice), essential for CSR to IgE [18]. High-affinity IgE antibodies, crucial for allergic reactions, arise from somatic hypermutation (SHM) and affinity maturation of B cell precursors within germinal centers (GC) [37].

**Figure 2:**
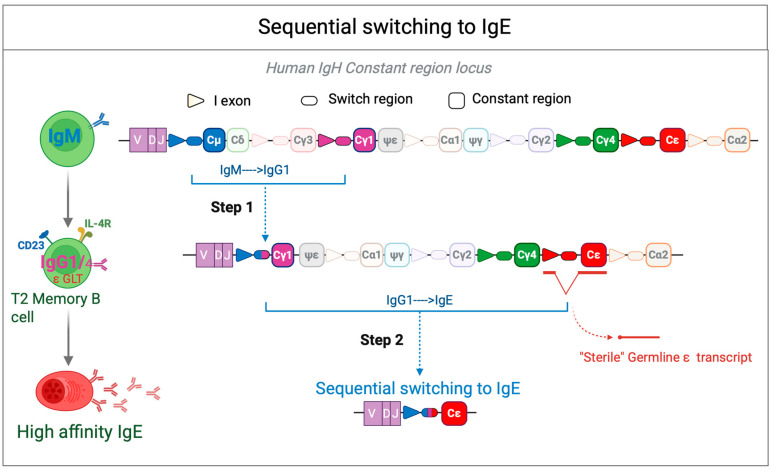
Sequential switching to IgE. Sequential class switching to IgE begins with the generation of IgG cells through recombination between Sμ and Sγ (typically Sγ1), which creates an Sμ-Sγ hybrid region. These IgG cells can then undergo affinity maturation within the germinal center (GC). Upon subsequent activation, a second recombination event may occur between Sμ-Sγ and Sε, leading to the generation of IgE-producing cells. The resulting Sμ(Sγ)-Sε hybrid region may either retain (Sγfl) or lose (Sγ”) Sγ sequences. The sterile epsilon (ε) germline transcript (GLT) is a noncoding RNA precursor transcribed from the immunoglobulin heavy chain (IGH) locus before CSR to IgE. This transcript is induced by IL-4 and IL-13 via STAT6 signaling and plays a crucial role in IgE class switching by facilitating the accessibility of the ε switch (Sε) region to activation-induced cytidine deaminase (AID). The sterile ε GLT does not encode a functional protein but serves as a key regulatory element in the recombination process that leads to IgE production in B cells.

Our group and others have shown that high-affinity IgE plasma cells are mostly generated through sequential switching (μ→γ→ε), with an intermediate IgG1-expressing phase (Figure 2). SHM and maturation occur in IgG1^+^ cells before switching to IgE and differentiating into plasma cells [38]. Adoptive transfer of IgG1^+^ B cells from immunized mice led to high-affinity IgE upon challenge, while IgG1-negative cells did not [39]. Furthermore, mice lacking IgG1 failed to produce high-affinity IgE against the hapten 4-hydroxy-3-nitrophenyl acetyl (NP), even after repeated immunizations [38]. In contrast, direct class switching from IgM to IgE (μ→ε) leads to the generation of low-affinity, minimally mutated IgE antibodies. Interestingly, recent studies found that IgE from IgG1-deficient mice could still induce anaphylaxis depending on immunization route and antigen type [40,41], suggesting that alternative pathways can produce pathogenic IgE. Given the near absence of IgE memory B cells in mice, these responses likely arise from class-switched memory precursors (e.g., IgG or IgM). Even if lower-affinity IgE was generated in the IgG1-deficient, it could still have been able to mediate anaphylaxis if avidity was increased by greater IgE diversity and by the lack of IgG1 neutralizing antibodies that modulate antigen thresholds for mast cell activation.

Importantly, in the context of allergies, high-affinity IgE is a key driver of mast cell activation and anaphylaxis, leading to symptoms such as histamine release and anaphylaxis [37]. SHM analysis of IgE switch regions in mice has revealed Sγ1 remnants in bone marrow IgE plasma cells after secondary immunization, suggesting these cells originated from IgG1-expressing B cells [42]. More recently, Asrat et al. showed that chronic allergen exposure further enriches long-lived IgE plasma cells for Sγ1 remnants, linking them to durable, potentially anaphylactic IgE responses [43]. These findings support in mice that sequential switching is the main pathway generating pathogenic high-affinity IgE in allergic responses.

### 3.2. IgE Plasma Cells and Memory B Cells in Food Allergies

Food allergies are largely mediated by allergen-specific IgE, but the role of B cell memory in maintaining long-term IgE responses remains unclear. A major unanswered question is why some food allergies resolve while others persist. Gaining insight into how high-affinity IgE is maintained could help explain allergy persistence. Although food-specific IgG and IgA memory B cells have been identified in humans [44,45], functional IgE memory B cells remain elusive [46,47], making it difficult to fully understand the origins and maintenance of IgE-producing cells. Persistent IgE responses indicate underlying memory mechanisms, including inducible class switching and long-lived IgE-producing plasma cells [47,48]. Some studies showed that allergen-specific IgE can last for years without re-exposure [49], and secondary exposures in mice rapidly boost IgE levels [50,51]. Another study showed that chronic allergen exposure in mice generates long-lived IgE-secreting cells in the bone marrow [43]. Similarly, cases where allergies persist following bone marrow transplantation suggest that memory B cells and/or long-lived plasma cells can be transferred from donor to recipient, contributing to continued allergen-specific IgE production [52]. Additionally, repeated immunization increases IgE affinity [38,39,53], a hallmark of immunological memory [54].

Our studies showed that high-affinity IgE, primarily generated through sequential class switching from IgG1 memory B cells, resides outside germinal centers and displays features of plasma cells both at the phenotypic and transcriptional level [39], while GC-stage IgE cells are transient, do not form memory populations, and have reduced expression of surface immunoglobulin and costimulatory molecules [42,55]. As a result, high-affinity IgE B cells bypass the germinal center stage, differentiating directly into plasma cells, while GC-stage IgE B cells are short-lived and apoptotic. Mouse studies showed that high-affinity IgE memory arises from IgG1 memory B cells through sequential CSR to IgE-secreting plasma cells [38,53,56]. Transcriptomic profiling has shown that IgE plasma cells resemble plasmablasts and are slower to mature than IgG1 plasma cells [57], consistent with immature IgE+ cells observed in human circulation [46]. We and others described in mice that CD73+CD80+ IgG1 memory B cells give rise to high-affinity IgE, while CD73−CD80− subsets yield lower-affinity responses upon reactivation [53,58], and are more likely to differentiate into IgG1 GC cells [53]. In humans, CD80+ memory B cells have strong potential for plasma cell differentiation [59]. Recently, our group [60,61] and others [62] have identified a type 2–polarized IgG1 and IgG4 memory B cell in allergic individuals that express IL-4R, CD23, and germline *IGHE* and are primed to switch to IgE [60]. Mouse models showed that allergen-specific type 2 memory B cells arise after sensitization and require IL-4 [62], likely acting as precursors for IgE plasma cells.

Increased circulating IgE+ B cells and plasmablasts have been observed in allergic individuals [63]. Though food-specific IgE plasma cells in bone marrow are not yet confirmed, cat-specific IgE PCs have been found in the bone marrow of cat-allergic patients, which have been shown to induce anaphylaxis in humanized mice, confirming their pathogenic role [43]. In another study, single-cell RNA sequencing of circulating IgE+ B cells in peanut-allergic patients revealed that most were immature plasmablasts with affinity-matured somatic mutations that bind Ara h 2 with high affinity [46], showing convergent clonal evolution across individuals. Additionally, IgE+ cells with a naïve or memory-like phenotype exhibited reduced membrane IgE expression [46], aligning with earlier reports of low surface IgE and diminished expression of the BCR signaling component CD79b in IgE+ cells [64].

## 4. Food Allergy Treatments

Food allergies not only pose a risk of severe reactions but also impact quality of life nutrition and create significant financial strain, underscoring the urgent need for safe, effective therapies. Although diagnostic and management strategies have improved, no curative treatments currently exist. Nowadays, management strategies primarily focus on avoiding allergens and promptly using emergency medications. As a result, creating new therapeutic options to prevent allergic reactions—especially severe ones—has become a critical goal for both individuals with food allergies and the healthcare providers managing their care. To address this, various therapeutic approaches are being explored, which will be discussed in this section.

### 4.1. Anti-IgE Targeted Immunotherapy

IgE-mediated food allergies develop when individuals produce IgE antibodies targeting specific food protein epitopes or food compounds. Given this mechanism, therapies that block IgE have become a rational approach for treating one or multiple IgE-mediated food allergies. In the early 2000s, a first-in-human study of anti-IgE therapy using Tazilumab (TNX-90) showed an increased threshold for peanut reactivity, though the drug was not pursued further [65,66]. Tazilumab is a humanized IgG1 monoclonal antibody that binds to an epitope on the Cε3 domain of IgE, preventing its interaction with FcεRI receptors on mast cells and basophils. Subsequent anti-IgE drugs, such as Omalizumab (Xolair) and Ligelizumab (developed by Novartis), have since been developed.

**Figure 3:**
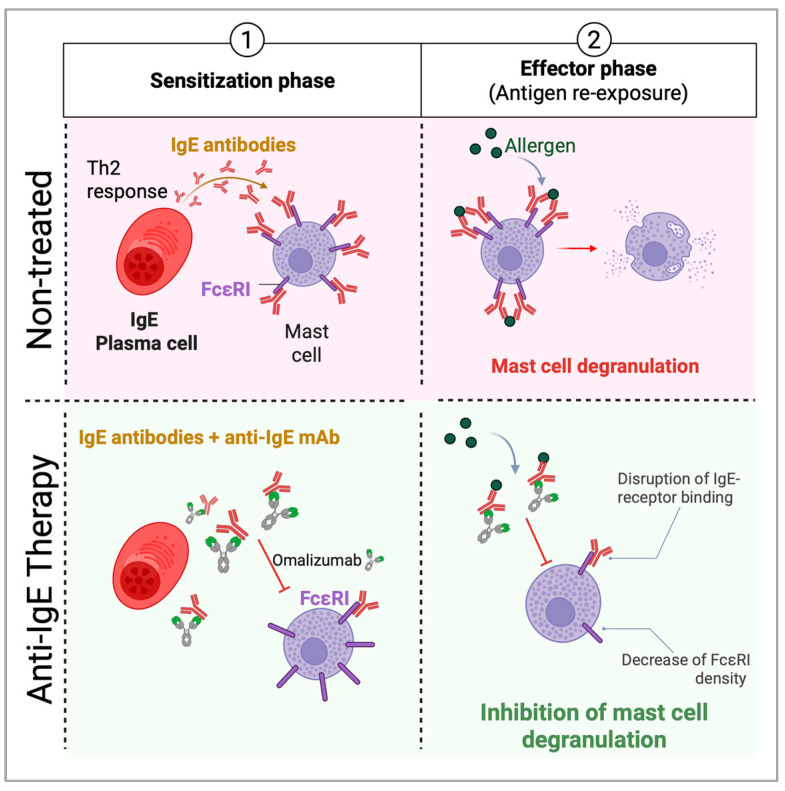
Anti-IgE targeted therapies mechanism of action on food allergic individuals. Epithelial cells release alarmins (IL-25, IL-33, TSLP), activating DCs to drive type 2 immunity. DCs capture allergens and present them to naïve T cells, promoting a Th2 response. Plasma cells produce IgE, binding FcϵRI on mast cells and basophils. Re-exposure (effector phase) triggers degranulation and anaphylaxis. Anti-IgE monoclonal antibodies, such as Omalizumab, bind to the Fc region of free IgE, preventing its interaction with FcεRI receptors on mast cells and basophils and decreasing the density of the FcεRI. This reduces cell activation and degranulation, thereby lowering the release of histamines, leukotrienes, and other inflammatory mediators. Additionally, over time, anti-IgE therapy downregulates FcεRI expression on effector cells, further dampening allergic responses.

Omalizumab is currently the only approved anti-IgE monoclonal antibody for clinical use, indicated for asthma, chronic spontaneous urticaria (CSU), nasal polyps, and, since February 2024, food allergy [66]. Omalizumab binds circulating free IgE at the Cε3 domain, blocking its ability to bind FcεRI on basophils and mast cells, thereby reducing mediator release and allergic symptoms [67] (Figure 3). The first randomized controlled trial (RCT) testing Omalizumab for food allergy was published by Sampson et al. in 2011 [68]. This small phase 2 trial showed that, after 24 weeks, participants receiving Omalizumab experienced an 80.9-fold increase in tolerated peanut dose compared to a 4.1-fold increase in the placebo group (*p* = 0.054). Although the study was terminated early due to severe reactions during the oral food challenges, initial results indicated that Omalizumab might raise the threshold of reactivity in peanut-allergic patients. Since then, other studies have supported the potential of Omalizumab as both a standalone therapy and in combination with oral immunotherapy (OIT) [66]. The FDA’s 2024 approval was based on data from the OUtMATCH trial (NCT03881696), which evaluated Omalizumab as monotherapy and as an adjunct to multi-allergen OIT in children and adults with food allergies [66,69]. In this trial, 58% of participants received subcutaneously a median dose of 300 mg every 2 weeks, whereas the median dose was 225 mg in the 42% of participants who received Omalizumab every 4 weeks.

Ligelizumab has also been investigated in clinical trials for asthma and patients with antihistamine-refractory chronic spontaneous urticaria (CSU) but has not outperformed Omalizumab [70]. These studies have shown that although targeting IgE, their clinical effects differ. A phase 3, multicenter, randomized, DBPC trial of Ligelizumab for peanut allergy monotherapy (NCT04984876) ended in 2023. However, a long-term extension study (NCT05678959) is underway, in which participants receive 120 or 240 mg of Ligelizumab subcutaneously every four weeks for up to three years to assess its long-term safety and effectiveness in peanut allergy [66]. As anti-IgE therapies transition into clinical use for food allergies, several key questions remain: optimal dosing and onset, safety in individuals with high baseline IgE, limited monotherapy data, and how Omalizumab and Ligelizumab compare in terms of safety and efficacy. Overall, anti-IgE therapy now offers a treatment option for some individuals with IgE-mediated food allergies, though it may not be suitable for all.

### 4.2. Allergen-Specific Immunotherapy (AIT)

Developing effective therapies to prevent allergic reactions, especially severe ones, remains a top priority for both patients and healthcare professionals managing food allergies. In this regard, allergen-specific immunotherapy (AIT) has emerged as a disease-modifying approach aimed at inducing immune tolerance to food allergens. AIT can be administered through various routes, including oral (OIT), sublingual (SLIT), and epicutaneous (EPIT). Subcutaneous immunotherapy (SCIT) is no longer commonly used for treating food allergies—especially to peanuts—because of its high risk of systemic allergic reactions and limited therapeutic benefit [71,72]. OIT involves swallowing allergen doses, while SLIT requires holding the allergen under the tongue before ingestion. Though OIT is associated with more adverse events than SLIT or EPIT, it also achieves higher rates of desensitization and is the most widely used method. EPIT, delivered through an allergen-containing patch applied to the skin, offers an alternative route to immune modulation. Despite differences in administration, all these approaches aim to promote desensitization through sustained allergen exposure [72,73]. Among them, OIT is the most used in clinical trials and has stronger evidence of its effectiveness and safety for food allergies, although it requires dose adjustments to maintain safety [74].

Oral immunotherapy (OIT) has become a major focus of research and clinical practice for food allergies, particularly for peanut, cow’s milk, and egg. It has been administered using various forms, including native foods, flours, powders, and commercial formulations. Among these, peanut OIT has seen the most rapid advancement in recent years and is now increasingly implemented in clinical settings, despite significant variation in dosing protocols and practices [75]. The efficacy in clinical trials has been defined by the induction of a desensitized state. OIT is not a curative approach but aims to induce a desensitized state, increasing the threshold for allergic reactions during treatment to reduce the likelihood and severity of reactions following accidental exposure to an allergen. The American Academy of Allergy, Asthma and Immunology (AAAAI) defines desensitization as “the improvement in food challenge outcome following therapy, dependent on continued allergen exposure”. In some cases, OIT may lead to sustained unresponsiveness (SU). This concept, introduced by Burks et al. [76], refers to “maintaining protection achieved through therapy and is not reliant on ongoing exposure”.

Currently, the Allergenic Products Advisory Committee of the U.S. Food and Drug Administration (FDA) has approved Palforzia (AR101, Aimmune Therapeutics), the first standardized peanut OIT product, which delivers measured doses of peanut protein. Additional studies demonstrated the efficacy of OIT in desensitizing children with other common food allergies in addition to peanuts [77]. The PALISADE study (NCT02635776) was a phase 3 clinical trial of Palforzia conducted for 12 months with food challenges at entry and exit. This study showed that AR101 increased the amount of peanut protein that highly allergic children and adolescents could consume without symptoms and reduced reaction severity during food challenges compared to placebo [78]. RAMSES (NCT03126227), another phase 3, placebo-controlled trial, evaluated Palforzia in real-world clinical settings and supported the efficacy and safety findings from PALISADE [79]. Palforzia received initial approval for use in children aged 4 to 17 to help lower the risk and severity of allergic reactions, including anaphylaxis, following unintentional peanut exposure. Recently, the FDA extended approval for Palforzia to include children aged 1 to 3, based on the POSEIDON study (NCT03736447), a phase 3 trial conducted across multiple centers in the U.S. (14 sites) and 9 sites in Europe (the United Kingdom, France, and Germany) [80].

An ongoing trial, the Small Children Oral Immunotherapy (SmaChO) study (NCT04511494) [81] is evaluating the efficacy and safety of a slower up-dosing regimen and lower maintenance dose over three years in young children aged 1–3, followed by a period of 4 weeks of peanut avoidance. Interim results from the first year suggest a high rate of desensitization with a favorable safety profile. While most OIT studies target patients with low reaction thresholds (≤100 mg of peanut protein), data are limited for individuals who only respond to higher doses (over 100 mg). The CAFETERIA trial (NCT03907397), a phase 2 study led by Sicherer et al. [82], focused on 73 children (4 to 14 years old) who were high-threshold responders—children who reacted only after consuming more than the equivalent of half a peanut. In this group, OIT was safe and effectively increased the reaction threshold, with a durable response in many participants after discontinuing treatment. Nevertheless, long-term outcomes and applications to other allergens remain under investigation.

Despite the promising data, after achieving SU, it remains unclear whether SU leads to lasting tolerance or to a return to allergy in the absence of exposure. Most studies report SU rather than true tolerance [75], but the ultimate goal is to ensure long-term tolerance, where individuals remain non-reactive to the allergen indefinitely and can safely incorporate the allergen into their diet. However, guidance is lacking on how to reintroduce allergens like peanuts after SU is achieved. In a recent long-term follow-up study, Lee et al. [83] tracked 44 children who passed the SU food challenge for peanuts. Over an average follow-up of 552 days (range: 146–1588 days), all continued to eat peanuts in various quantities and frequencies, with no reported adverse reactions. While this outcome is promising, further long-term research is needed to confirm whether SU can lead to lasting tolerance.

## 5. Changes in Allergen-Specific Immunity After AIT

As outlined earlier, OIT remains the sole treatment capable of altering the course of allergic diseases by fostering both clinical and immunologic tolerance. OIT induces desensitization by increasing the reaction threshold during therapy, and in some cases, it leads to remission, where patients remain non-responsive after stopping treatment. However, the mechanisms underlying long-term tolerance remain unclear. Understanding the molecular and cellular factors that determine lasting protection is crucial for guiding immunotherapy decisions.

### 5.1. Immunophenotypic Changes in Circulating Allergen-Specific Memory B Cells

Although food-specific memory B cells and IgE plasma cells are responsible for maintaining the IgE response, their scarcity poses challenges for detailed investigation. Only a limited number of studies have examined allergen-specific B cells—especially memory subsets—in the context of food allergy. These cells are infrequently found in the peripheral blood of allergic individuals but become more abundant during oral immunotherapy (OIT), allowing for their identification and analysis in children undergoing treatment [44,45,84].

Hoh et al. [45] used fluorescently labeled peanut allergens, Ara h 1 and Ara h 2, to examine peanut-specific B-cell populations before and during OIT. They observed a marked increase in class-switched memory B cells specific to the allergen. Similarly, Patil et al. [44] utilized fluorescent Ara h 2 multimers to monitor Ara h 2–specific memory B cells in participants of a randomized, open-label peanut OIT trial (PNOIT; NCT01324401). Their results showed that these memory B cells emerged early in treatment but were transient. These observations aligned with earlier findings by Vickery et al. [85] using a peptide microarray–based assay. Notably, the expansion of Ara h 2–specific memory B cells peaked around the seventh week of therapy, coinciding with increased levels of Ara h 2–specific IgA, IgG, and IgG4 antibodies. Importantly, immunoglobulins produced by these circulating Ara h 2–specific B cells displayed evidence of affinity maturation. In addition, allergen-specific IgG4 antibodies were found at higher levels in OIT-treated individuals, reinforcing the association between IgG4 responses and clinical improvement during immunotherapy [86]. Most peanut-reactive B cells expressed class-switched, somatically mutated antibodies, and their frequency increased with immunotherapy. The findings indicate that OIT drives the production of highly mutated allergen-specific IgG4, which likely contributes to its therapeutic effect [45].

Two recent studies [84,87] have explored how allergen-specific antibody responses evolve during AIT, with a focus on memory B cell changes linked to desensitization in bee venom (BV) and cow’s milk allergy (CMA). McKenzie et al. [87] investigated memory B cells in 26 individuals with a history of systemic reactions to bee stings and elevated serum levels of BV-specific IgE (>0.35 kU/L), analyzing samples taken before and up to 63 days after beginning SCIT for BV allergy. Prior analysis revealed that BV SCIT led to an expansion of circulating IgG+ memory B cells targeting Api m 1, with notable phenotypic changes such as increased CD23 (recently described as a marker for type 2 memory B cells [60,61,62]) and CD29 (or integrin β1, part of very late antigen 4 (VLA-4), which mediates leukocyte adhesion and may regulate BCR signaling), but not IL-13Rα, and a shifted BCR epitope specificity after BV SCIT. After two months of BV SCIT, enhanced CD29 expression in Api m 1–specific memory B cells mirrored similar observations in Lol p 1–specific memory B cells following SLIT for ryegrass allergy [88]. Moreover, a shift in antibody isotype distribution toward IgG2 and IgG4 was noted among Api m 1–specific memory B cells following treatment, while the total memory B-cell pool remained unchanged. Like in ryegrass pollen SLIT, BV SCIT increased CD23+ allergen-specific memory B cells—suggestive of type 2 memory B cells expressing IgG with potential to switch to IgE. Although this may appear contradictory, their altered phenotype may contribute to AIT-induced desensitization, including the generation of plasma cells that produce IgG1, IgG2, or IgG4.

In the context of cow’s milk allergy, Satitsuksanoa et al. [84] conducted a study involving 13 children undergoing OIT and 9 children who naturally outgrew their allergy. Throughout 12 to 15 months following the initiation of OIT, they found an increase in the frequency of allergen-specific B cells, particularly those producing IgG4—a feature observed both in children achieving remission and those with natural tolerance (NT). This increase was associated with a reduced IgE/IgG4 ratio, suggesting a shift toward a more tolerogenic immune profile. The study provided a detailed analysis of Bos d 9–specific B cells, assessing gene expression, protein secretion, and antibody production in children who were desensitized, in remission, or had naturally outgrown CMA. Principal component analysis (PCA) of the top 200 differentially expressed genes (DEGs) revealed distinct clustering of four groups: pre-OIT, post-OIT (desensitization and remission), and NT. Post-OIT, desensitized and remission patients exhibited divergent B cell responses.

Allergen-specific B cell responses diverged after OIT, with unique transcriptomic changes distinguishing desensitization from remission. In remission, B cells showed downregulation of genes involved in activation pathways—such as those related to antigen recognition, BCR signaling, cytokine signaling (*IL21R*), chemotaxis, and differentiation—as well as increased expression of regulatory genes like *IL10RA*. Interestingly, TLR4 expression was also elevated in the remission group. While *IGHG2* was the only antibody-related gene significantly upregulated in desensitized individuals, remission was associated with increased expression of genes encoding multiple immunoglobulin isotypes, including IgG4, IgG1, IgA1, IgA2, and IgD. These antibody patterns—particularly the rise in IgG4—have consistently been linked to the development of clinical tolerance and remission following OIT for various food allergens [84]. While remission-specific B cells underwent significant transcriptomic changes post-OIT, they did not fully mirror NT [84]. These findings suggest that desensitization may be a step toward remission and long-term tolerance. Overall, this study concluded that allergen-specific B cells undergo additional transcriptomic changes beyond those seen in the desensitized state in individuals who achieve remission following OIT, including further silencing of genes involved in B cell activation. Compared to desensitized individuals, those in remission showed a more pronounced downregulation of gene expression pathways related to B cell suppression, while allergic individuals showed a pro-inflammatory profile driven by type 2 cytokine-related genes. Children who naturally outgrew food allergies shared some immune features with those in remission, though key differences existed. In NT, B cells differentiated into pre-PCs and marginal zone B cell types, expressing more innate immune receptors. However, remission was characterized by the expression of B regulatory (Breg) cell-related genes, with increased expression of *IGHG4*, *IL10RA*, and *TGF-β* genes, which have been linked to immune tolerance.

These findings emphasize that allergen-specific B cells undergo active modulation during both OIT and NT, with their distinct gene expression profiles playing a key role in establishing and sustaining immune tolerance to food allergens.

### 5.2. Serum Allergen-Specific Immunoglobulin Changes

Studies conducted on BV and ryegrass pollen allergies after SCIT indicate that both immunophenotypic and serum allergen-specific immunoglobulin changes occur after AIT [87,88], shifting isotypes from allergen-specific IgE to IgG4. Although the protective role of IgG4 is well established in BV SCIT, it cannot be readily applied to other allergy types because of several differences, such as the nature of the antigens and tissue entry. However, findings from a one-year follow-up of the POISED trial (Peanut Oral Immunotherapy Study: Safety, Efficacy, and Discovery; ClinicalTrials.gov ID: NCT02103270) demonstrated that peanut OIT induces significant changes in allergen-specific antibodies—specifically, a decrease in peanut-specific IgE and an increase in IgG4—both of which were associated with achieving SU [89]. In a study on CMA, Satitsuksanoa et al. [84] performed a detailed analysis of B cells specific to Bos d 9 (αS1-casein), the primary allergen in cow’s milk, to investigate how antigen-specific B cell responses correlate with the development of remission following OIT and in children spontaneously outgrowing CMA through natural outgrowth (NT). They observed that food-allergic individuals had relatively high allergen-specific IgE production, together with allergen-specific IgG1 and relatively low allergen-specific IgG4 antibodies. After OIT, allergen-specific IgG4 became the predominant allergen-specific antibody isotype, with very low allergen-specific IgG1, and no significant change was observed in allergen-specific IgE. Interestingly, allergic individuals before OIT showed the highest IgE to IgG4 ratio, whereas the NT group exhibited the lowest. Additionally, IgG4 production by B cells did not significantly differ between the desensitized and remission groups. In contrast, NT individuals displayed a strong allergen-specific IgG4 response alongside relatively low levels of allergen-specific IgE, indicating that while overlapping, the mechanisms driving NT and OIT-induced tolerance are not entirely the same.

Studies on OIT for peanut allergy have shown that allergen-specific IgG, IgA, and IgG4 levels rise in all subjects but persist only in those with sustained responses after treatment. In these individuals, reduced allergen-specific IgE levels are accompanied by decreased sensitivity to allergen-induced basophil activation [90,91]. Findings from the Learning Early About Peanut Allergy (LEAP) trial also support the idea that early exposure to peanuts can help prevent peanut allergy by promoting the development of allergen-specific IgG4 antibodies. In contrast, individuals who avoided peanuts were more likely to develop peanut-specific IgE responses [92,93,94]. All these data suggest that the composition of allergen-specific antibody repertoires shaped during AIT is closely linked to its clinical effectiveness.

In contrast to IgG, the role of IgA in food allergy remains less well understood and has received comparatively little research attention. Secreted IgA (SIgA) is believed to mitigate allergic responses by preventing food antigen absorption in the gut. Studies have linked low IgA levels to atopic disorders; for instance, reduced salivary secreted IgA has been linked to sensitization, allergic rhinitis, and atopic dermatitis, while higher levels are associated with protective effects, particularly in infants with allergic predispositions. SIgA has been shown to inhibit chemotaxis of immune cells such as neutrophils, eosinophils, and monocytes, and to reduce IgE-driven histamine release [95,96], so high mucosal SIgA levels may block allergen-IgE interactions and help prevent allergic symptoms [34]. Similarly, higher total SIgA in feces has been associated with reduced risk of atopy, reinforcing the idea that IgA deficiency may contribute to allergy development [10].

To better understand antigen-specific antibody responses at the oral mucosa during peanut oral immunotherapy (PnOIT), Smeekens et al. [97] measured salivary levels of peanut-specific and total IgG4 and SIgA in children enrolled in the Immune Tolerance Network’s IMPACT study (ClinicalTrials.gov ID: NCT01867671), a phase 2 trial of PnOIT. Children between 12 and <48 months old were randomized to receive either peanut or placebo OIT for 134 weeks. Those receiving OIT exhibited substantial increases in salivary IgG4 and SIgA against peanuts, pointing to mucosal immune activation and suggesting saliva as a promising, non-invasive source for immunological biomarkers. Further evidence for a protective role of SIgA comes from research by El Ansari et al. [98], who found that SIgA antibodies can bind mast cells and basophils in mice. Their study demonstrated that allergen-specific SIgA was capable of blocking IgE-mediated activation of these effector cells in both human and mouse models. Specifically, SIgA inhibited peanut-triggered activation of basophils taken from a peanut-allergic individual [98]. These results suggest that allergen-specific SIgA plays a role in modulating mast cell and basophil responses, pointing to its potential function in preserving immune balance within mucosal tissues. In addition, breast milk contains allergen-specific SIgA and IgG, with IgA suggested to play a protective role modulating CMA. Jarvinen et al. observed that maternal avoidance of cow’s milk was linked to lower mucosal SIgA levels and an elevated risk of CMA in infants [99]. Moreover, total SIgA concentrations in breast milk have been inversely associated with early-onset atopic dermatitis, highlighting the possible role of maternal SIgA in fostering tolerance in infants [3].

Nevertheless, several studies have failed to consistently link food-specific IgA levels with the development of natural tolerance (NT) or allergic outcomes, suggesting that IgA alone may not serve as a dependable biomarker. In one study, Zhang et al. [100] evaluated peanut-specific IgA responses in mice after daily peanut exposure and found negligible levels of allergen-specific IgA even after 42 days. Similarly, Liu et al. [101] reported that fecal peanut-specific IgA in 441 atopic children did not predict future allergy protection. These findings challenge the role of IgA in tolerance, though variations in timing, sample location, and patient atopic status may influence the results. Additional research reported no correlation between gut-derived peanut-specific SIgA and subsequent allergy or tolerance in a group of atopic infants. Interestingly, plasma peanut-specific IgA levels were elevated in allergic compared to non-allergic children. Likewise, egg-allergic children with higher levels of gut egg white–specific SIgA (egg white whole extract, including all proteins) did not show improved chances of predicting allergy resolution. Moreover, peanut-specific IgA epitope recognition was similar in both allergic and non-allergic infants [101]. These observations challenge the notion that antigen-specific IgA consistently contributes to immune protection. Overall, findings across studies remain inconclusive, possibly due to compartment-specific differences (e.g., secretory and serum) in IgA responses. More rigorous and standardized research is necessary to better define IgA’s role in allergy and its potential as a clinical biomarker for diagnosis or prognosis.

**Figure 4:**
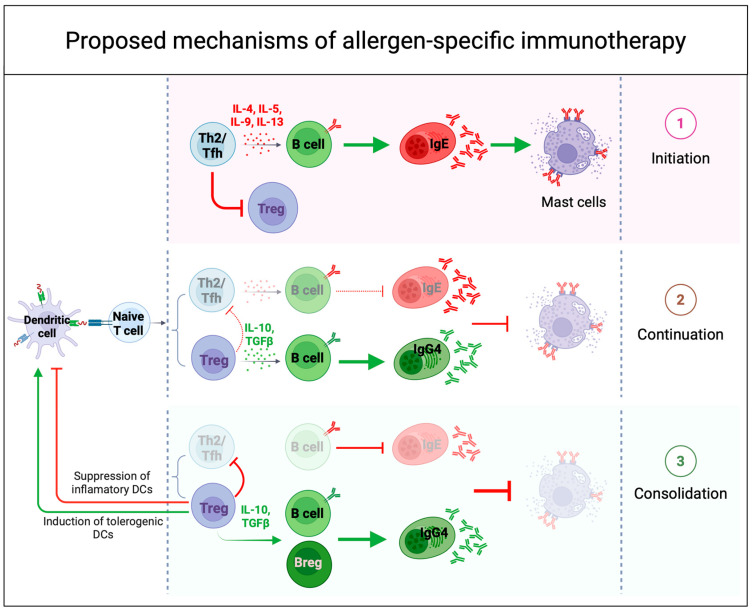
Proposed mechanisms of immune tolerance to allergens during successful allergen-specific immunotherapy (AIT). Successful AIT induces immune tolerance through several mechanisms that modulate both innate and adaptive immune responses. (**1**) During the initiation phase of OIT, low-dose allergen exposure amplifies a type 2 effector response and inflammatory B cell activity, while simultaneously inhibiting the early induction of regulatory T cells (Tregs) and leading to mast cell degranulation. (**2**) As OIT continues with increasing doses, chronic allergen exposure induces a counterregulatory response, leading to reduced pro-inflammatory activity, increased IL-10 and TGFβ production, and a shift in the IgE/IgG4 ratio—hallmarks of desensitization. (**3**) With continued dosing, the immune system enters a consolidation phase, where prolonged antigen exposure promotes T cell exhaustion or deletion and redirects responses away from type 2 pathways. This phase may also reinforce Treg differentiation and B regulatory cells (Breg) induction, modulating dendritic cells (DCs) function and contributing to long-term immune tolerance and sustained unresponsiveness.

Oral immunotherapy has consistently achieved desensitization in food allergy patients completing clinical trials, with some also attaining sustained unresponsiveness. While these outcomes are linked to distinct changes in allergen-specific immune responses, a comprehensive understanding of OIT’s mechanisms remains incomplete. Kulis et al. [102] proposed a mechanism of action for AIT based on existing evidence (Figure 4). Since allergen-specific TH2 and T follicular helper (Tfh) cells play a key role in allergic responses, the effectiveness of OIT likely depends on how antigen exposure modulates CD4+ T cell responses in both magnitude and polarization, influenced by dose and duration. Early low-dose allergen exposure (OIT initiation) may initially boost TH2/Tfh responses and inhibit regulatory T cell (Treg) development. They proposed that as the allergen dose increases during the escalation phase of the therapy, TH2 activity and clonal expansion decline, while IL–10–producing CD4+ T cells increase, promoting a shift toward immune regulation. This shift supports the production of allergen-specific IgG4 antibodies, which may help to neutralize IgE-mediated allergic reactions and may suppress the formation of new pathogenic TH2/Tfh cells. Despite these changes, an elevated frequency of allergen-specific CD4+ T cells remains, potentially explaining why clinical benefits can diminish or disappear if OIT is interrupted or discontinued prematurely. With ongoing dosing, the immune system enters a consolidation phase, where prolonged allergen exposure leads to T cell exhaustion or deletion and shifts responses away from TH2 pathways. This phase also supports Treg and Breg development, promoting long-term tolerance and sustained unresponsiveness.

In summary, successful desensitization following allergen immunotherapy (AIT) is associated with increased levels of allergen-specific total IgG, IgG4, and IgA antibodies, alongside a reduction in allergen-specific IgE. The rise in non-IgE antibody isotypes is strongly implicated in mitigating IgE-mediated allergic responses. Nonetheless, the precise immunological mechanisms by which IgG and IgG4 contribute to immune tolerance remain incompletely defined. It is still unclear to what extent these antibodies act through direct neutralization of allergens versus engagement of inhibitory Fcγ receptors. Furthermore, the utility of allergen-specific IgG or IgG4 as predictive biomarkers for long-term therapeutic success after OIT continues to require further investigation.

### 5.3. Allergen-Specific Isotypes Shifting After AIT: Do They Possess Neutralizing Capabilities?

Although the role of allergen-specific IgE in initiating allergic reactions is well established, the contributions of allergen-specific IgG and IgA antibodies are less well defined but increasingly recognized. Allergen-specific IgE, produced following sensitization, promotes allergic symptoms and enhances allergen uptake by immune cells. In contrast, IgG and IgA levels rise significantly following AIT, implying a potential protective effect by interfering with IgE-mediated mechanisms [3]. These antibodies may help promote tolerance by blocking allergen binding to IgE on mast cells and basophils, thus preventing their activation. Notably, AIT often induces allergen-specific IgG4, which has been shown to compete with IgE for allergen binding and is linked to long-term IgE-inhibitory activity after AIT, as seen in treatments for birch pollen allergy [87]. IgG1 and possibly IgG2 may also contribute to this process by either inhibiting IgE-allergen binding or engaging the inhibitory Fcγ receptors on immune effector cells. Furiness et al. [10] suggested that allergen-specific IgA and IgG generated during AIT can counteract IgE-driven mast cell activation by outcompeting IgE for allergen binding, thus hindering the formation of allergen-IgE complexes and reducing engagement with FcεRI and FcεRII on effector and B cells. This mechanism is supported by findings linking cat- and birch-specific IgG in cord blood to reduced allergy risk and by associations between low IgA levels and increased risk of cow’s milk allergy. In addition, in vitro studies indicate that high allergen doses can override this blocking effect, as seen in basophil activation tests [3].

Another proposed neutralizing mechanism involves IgG binding to inhibitory CD32b receptors (FcγRIIB, the low-affinity inhibitory receptor for IgG), while IgE binds FcεRI on mast cells, reducing degranulation, though this is mainly observed in artificial models. AIT also promotes the secretion of regulatory cytokines such as IL-10 and TGF-β from monocytes and T cells, encouraging the development of regulatory T cells and promoting antibody class switching toward IgG and IgG4 isotypes. These changes can downregulate FcεRI expression and reduce effector cell activation, contributing further to immune tolerance [3]. The dynamics between allergen-specific IgE and other isotypes vary by allergen type. AIT typically shifts the immune response away from a Th2-dominated profile toward a more regulatory state, characterized by reduced Th2 cytokine production, decreased eosinophil infiltration, and fewer allergic symptoms.

Recent findings suggest that allergen-specific IgG antibodies generated during OIT can actively suppress basophil activation in IgE-sensitized individuals, thereby contributing to a clinically nonreactive state [98,103]. Most studies conducted to assess the allergen-specific isotype composition before and after OIT resulted in a decrease in allergen-specific IgE and an increase mostly in IgG4. A growing interest in understanding the biological function of these allergen-specific IgG4 antibodies has led to deeper studies, revealing some neutralization capabilities. James et al. [104] found that long-term tolerance after grass pollen immunotherapy for allergic rhinitis is associated with persistent blocking antibodies. They showed this by testing patient sera for IgG-dependent inhibition of IgE-allergen binding to B cells. Despite a decline in allergen-specific IgG1 and IgG4 levels post-treatment, inhibitory IgG activity remained unchanged, suggesting a role in sustained clinical tolerance. This data is supported by Nouri-Aria et al. [105], who determined that the inhibitory activity resided within the IgG4 fraction of the post-AIT serum. Therefore, a growing body of evidence supports a link between increased levels of allergen-specific IgG, particularly IgG4, and clinical improvement following AIT. For instance, enhanced IgG4 “blocking” activity has been associated with reduced symptoms in patients receiving grass pollen immunotherapy, suggesting that IgG4 may play a protective role by interfering with IgE-allergen interactions.

Additional support for the inhibitory role of IgG4 comes from studies where depletion of peanut-specific IgG4 from the plasma of individuals undergoing OIT led to diminished inhibition of mast cell activation [106]. In the same study, some samples had only a partial effect after IgG4 depletion, suggesting that other isotypes—such as peanut-specific IgA—might also contribute by competing with IgE for allergen binding. In a separate study, Keswani et al. [107] described the existence of neutralizing Ara h 2-specific IgG4 antibodies detected at high levels only in patients with sustained unresponsiveness after OIT for peanut allergy. These antibodies inhibit IgE-mediated degranulation induced by peanut or Ara h 2, highlighting their use as clinical biomarkers of sustained efficacy after peanut OIT. These studies strongly suggest that the shift toward IgG4 isotypes after successful AIT is driven by their ability to neutralize allergen-specific IgE. Interestingly, Strobl et al. [108] examined the relative contributions of IgG1 and IgG4 to IgE-blocking activity over three years of birch pollen AIT. Their findings showed that while IgG1 dominated in the early phase, IgG4 became the predominant blocking isotype later in treatment—despite both maintaining strong allergen-binding avidity. Supporting this, Smith et al. [109] generated human IgE monoclonal antibodies (mAbs) using a cell culture method. These mAbs were characterized through allergen binding assays and functional assays in vivo and in vitro. In addition, Croote et al. [110] engineered IgG4 versions of key Ara h 2-binding antibodies that could significantly suppress mast cell degranulation in vitro, even when sensitized with polyclonal IgE from unrelated peanut-allergic donors. These results highlight the adaptability and central role of IgG4 in mediating immune modulation during AIT.

These findings raise the possibility that such neutralizing antibodies could serve as useful biomarkers. However, a deeper understanding of the immune mechanisms underlying AIT is needed to improve therapy and identify reliable predictors of success. In this regard, Sackesen et al. [111] and Suprun et al. [112] offered valuable insights into how IgE and IgG4 antibody repertoires are associated with disease phenotypes and can help predict clinical outcomes following OIT for milk and peanut allergies. Using a high-throughput Luminex-based peptide assay (LPA), Sackesen et al. [111] identified distinct IgE/IgG4 epitope-binding patterns across CMA phenotypes, demonstrating its potential for predicting CMA severity. Similarly, Bead-Based Epitope Assay (BBEA)—a highly reliable and sensitive method for detecting epitope-specific antibodies, tested in peanut allergy [112]—showed that epitope-specific IgE is a valuable biomarker for food allergy severity and phenotypes, while IgE/IgG4 quantification provides deeper insights into allergic sensitization. Additionally, Ho et al. [113] investigated whether pre-challenge levels of peanut-specific antibodies in saliva could be used as non-invasive biomarkers to assess allergy severity and threshold in a study involving 127 children undergoing double-blind, placebo-controlled peanut challenges. They found that higher saliva peanut-specific IgE levels were associated with increased odds of likely true peanut allergy and respiratory symptoms. Conversely, higher peanut-specific IgG4 or a higher peanut-specific IgA:IgE or IgG4:IgE ratio in saliva correlated with lower odds of severe reactions and lower likelihood of peanut allergy, underscoring the potential utility of saliva antibody profiling in clinical evaluation.

Current treatment options for food allergies are limited by their slow onset of action and the requirement for continuous administration. Individuals with peanut allergies, in particular, would benefit from therapies that can provide rapid protection from accidental exposure while reducing the likelihood of adverse events. Conventional allergy tests typically measure allergen-specific IgE titers, but they fail to consider clonality or affinity. As a result, these tests may overestimate clinical allergy severity and poorly correlate with allergic reactions. Croote et al. [110] investigated the molecular features of human monoclonal IgE antibodies involved in peanut allergy, including their sequences, binding strength, and clonality. Using the SEQ SIFTER single-cell RNA sequencing platform developed by IgGenix, Inc. (UnitedHealthcare Group, Salt Lake City, UT, USA) [46,114], they identified a convergence of high-affinity IgE monoclonal antibodies directed against a recurring DPYSPS motif unique to the major peanut allergen Ara h 2—consistent with patterns seen in peanut-specific memory B cells [60,115]. These high-affinity IgE mAbs could independently trigger mast cell degranulation and systemic anaphylaxis in humanized mouse models [110]. Based on these findings, the team engineered IGNX001, a therapeutic composed of two high-affinity IgG4 monoclonal antibodies derived from IgE sequences. This IgG4-based therapy effectively blocked mast cell activation in vitro and prevented anaphylaxis in a peanut allergy mouse model following intragastric peanut challenge. Such advances represent a promising step toward more targeted and rapid-acting treatments for food allergies.

## 6. Conclusions

Food allergies are sustained by immunological memory. Non-IgE memory B cells, mainly of the IgG1 subclass, and IgE plasma cells contribute to the persistence of IgE production. Allergen immunotherapy (AIT) offers the only disease-modifying treatment thus far, inducing desensitization and, in some cases, long-term remission by altering the immune profile. The increase in IgG4 and IgA levels and the decrease in IgE are key indicators of therapeutic success, with a strong IgG4 increase and the formation of neutralizing IgG4 antibodies linked to remission. In contrast, the role of allergen-specific IgA remains unclear. Biomarkers such as allergen-specific IgG4 and IgA antibodies and changes in the phenotype of memory B cells show promise for monitoring treatment progress, though further research is necessary to validate their predictive value for long-term tolerance. A better understanding of the mechanisms behind IgG1 memory B cell responses and their role in food allergy persistence, as well as the unique characteristics of IgE plasma cells, will be critical for the development of targeted therapies.
